# Periodontitis is associated with an increased risk for proximal colorectal neoplasms

**DOI:** 10.1038/s41598-019-44014-8

**Published:** 2019-05-17

**Authors:** Gun Woo Kim, Young-Sang Kim, Soo Hyun Lee, Seung Geon Park, Duk Hwan Kim, Joo Young Cho, Ki Baik Hahm, Sung Pyo Hong, Jun-Hwan Yoo

**Affiliations:** 1 Digestive Disease Center, CHA Bundang Medical Center, CHA University, 59 Yatap-ro, Bundang-gu, Seongnam 13496 South Korea; 2 Department of Family Medicine, CHA Bundang Medical Center, CHA University, 59 Yatap-ro, Bundang-gu, Seongnam 13496 South Korea

**Keywords:** Risk factors, Colorectal cancer

## Abstract

Interval colorectal cancers detected after colonoscopy are known to be highly associated with proximal colorectal neoplasms (CRNs). This cross-sectional study investigated whether periodontitis could be a risk factor for proximal CRNs in healthy individuals. A total of 2504 subjects who received a colonoscopy and dental exam were enrolled in this study. We divided the subjects into the periodontitis group (n = 216) and the control group (n = 2288). The periodontitis group was defined as subjects who had one or more teeth with a probing pocket depth (PPD) ≥4 mm. The prevalence of proximal CRNs was significantly higher in the periodontitis group (25.0%) than in the control group (12.3%) (*P* < 0.001). Independent risk factors for proximal CRNs in the multivariate analysis were periodontitis, smoking, age, waist circumference, and triglycerides, and those for proximal advanced CRNs were periodontitis, age, and family history of CRC. However, periodontitis was not a risk factor for overall CRNs and advanced CRNs. Periodontitis was associated with an increased risk of proximal CRNs (odds ratio [OR], 1.525; 95% confidence intervals [95% CI], 1.071–2.172) and proximal advanced CRNs (OR, 2.671; 95% CI, 1.088–6.560). Periodontitis might be associated with proximal CRNs and proximal advanced CRNs.

## Introduction

Colonoscopy has been used for early detection and removal of premalignant lesions to reduce the mortality from colorectal cancers (CRCs). However, previous studies have reported that, after colonoscopy screening, there was a lower reduction in mortality from proximal CRCs than from distal CRCs^1–3^. The underlying reason for this finding could be attributed to a higher frequency of interval CRCs (CRCs detected after the index colonoscopy before the next recommended surveillance examination) in the proximal colon^3–7^. Most interval CRCs arise from missed lesions occurring more frequently in the proximal colon^5,7,8^. Proximal colorectal neoplasms (CRNs) are more often missed due to a flat and sessile appearance, worse bowel preparation in the proximal colon, and incomplete colonoscopy^1,9,10^. Moreover, proximal CRNs, which have been known to be biologically different from distal lesions, could result in a more rapid progression to CRCs. Several molecular features such as microsatellite instability (MSI)-high, CpG island methylation phenotype (CIMP)-high, and serrated pathway signature might have a role in this progression^3,11–13^. Therefore, meticulous inspection of the proximal colon is required in subjects with a high risk for proximal CRNs.

Previous studies have shown that age, male sex, distal adenoma, body mass index (BMI), and distal hyperplastic polyps may be predictive of proximal CRNs^14–17^. Confining the analysis to the proximal CRNs without distal adenomatous findings, the risk factors were age, smoking, and a family history of CRC (FH of CRC)^18^. However, compared to many studies regarding predictive factors for overall CRNs^19^, few studies have focused on those for proximal CRNs. In addition, most risk factors which are considered as predictive for proximal CRNs overlap with those for overall CRNs. Therefore, these observations suggest that further studies are required to identify the specific predictive factors for proximal CRNs, and the factors which are associated with biological or molecular features of proximal CRNs could be promising candidates.

Recently, an epidemiologic study reported that women with fewer teeth might be at a modest increased risk of proximal CRC, suggesting a potential role of oral health in colorectal carcinogenesis in the proximal colon^20^. Another study observed that periodontal disease was associated with increased CRC mortality^21^. Periodontitis is a chronic inflammatory disease caused by oral microorganisms and characterized by progressive destruction of the tooth-supporting apparatus leading to tooth loss^22^. Increasing evidence indicates that periodontitis is associated with several gastrointestinal cancers, occurring in the pancreas, esophagus, stomach, and colorectum^20,23–25^. Periodontal pathogens or their toxins might enter the blood and increase systemic inflammation which have a critical role in gastrointestinal carcinogenesis^21^. It is also possible that oral microbial dysbiosis caused by periodontitis might alter the gut microbiota by swallowed bacteria, and thus could have important implications for CRC development^20,26,27^. Several periodontal pathogens such as *Fusobacterium*, *Leptotrichia*, and *Campylobacter* have been linked to CRC^28,29^. In addition, some studies have shown that *Fusobacteria* is enriched in human CRNs and promote intestinal tumorigenesis by modulating the tumor-immune microenvironment^30–32^. Interestingly, *Fusobacterium* was detected markedly more in proximal CRNs than in distal CRNs^33–35^. *Fusobacterium* was more frequently detected in CIMP-high and MSI- high CRNs which increased gradually from the rectum to the ascending colon^35–37^. These findings may support biologically the plausible linkage between periodontitis and proximal CRNs.

A recent study showed that periodontitis defined by a self-report of oral health increases the risk of colorectal adenoma^38^. However, no study has ever investigated an association of proximal CRNs and periodontitis defined by objective criteria. Therefore, we investigated whether individuals with periodontitis might have a higher risk of proximal CRNs and proximal advanced CRNs.

## Results

### Baseline characteristics

**Figure 1:**
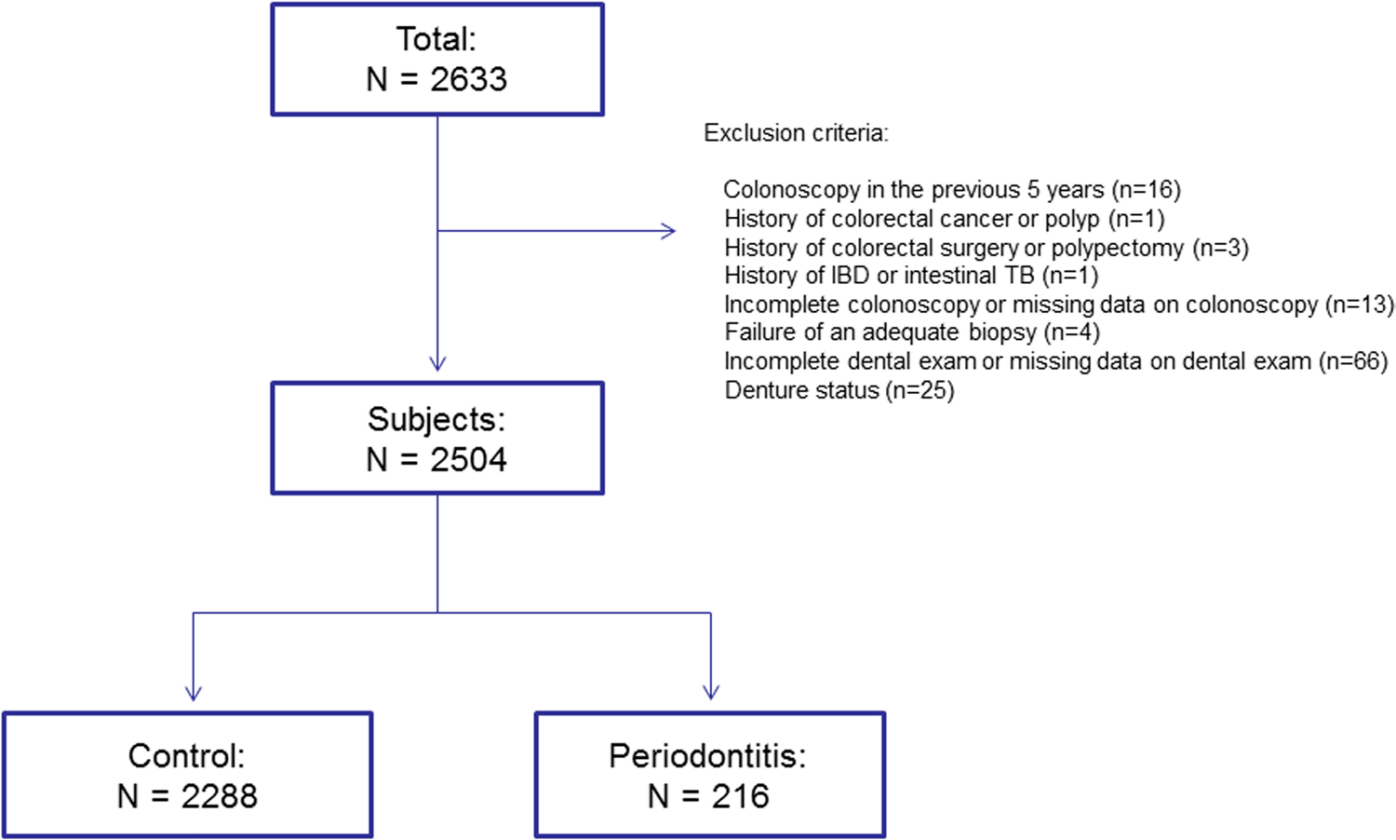
Flow diagram illustrating the exclusion of the study subjects from this analysis for the reasons indicated. Abbreviations: IBD, Inflammatory bowel disease; TB, Tuberculosis.

In total, 2504 subjects were included in the final analysis after the exclusions were complete (Fig. 1). Table 1 presents the baseline characteristics and the results of the colonoscopy for the subjects. The overall prevalence of periodontitis was 8.6% (216/2504). Compared to the control group, the periodontitis group was more likely to be men, had an older age, higher BMI, higher waist circumference, and lower HDL level, and had a higher prevalence of metabolic syndrome, hypertension, high fasting glucose, smoking (ever), and aspirin use. When comparing the results of the screening colonoscopy, the prevalence of CRNs (all) and proximal CRNs was significantly higher in the periodontitis group (CRNs [all]: 35.2% in the periodontitis group vs. 21.9% in the control group, *P* < 0.001; proximal CRNs: 25.0% vs. 12.3%, *P* < 0.001). The prevalence of advanced CRNs and proximal advanced CRNs was also significantly higher in the periodontitis group (advanced CRNs: 4.2% vs. 1.5%, *P* = 0.004; proximal advanced CRNs: 3.2% vs. 0.9%, *P* < 0.001). The clinicopathological characteristics of the CRNs detected are shown in Table 2. The number and size of the CRNs detected were significantly more and bigger in the periodontitis group than in the control group. The CRNs in the periodontitis group were more likely to be polypoid lesions. However, the degree of dysplasia and histology did not differ significantly between the control and periodontitis group. Table 3 presents the characteristics of the CRNs regarding location. A higher proportion of subjects in the periodontitis group had CRNs in the proximal AC (30.3% in the periodontitis group vs. 7.2% in the control group, *P* < 0.001), and TC (38.2% vs. 25.9%, *P* = 0.026). Compared to the control group, the periodontitis group was more likely to have CRNs in the proximal (71.1% in the periodontitis group vs. 56.0% in the control, *P* = 0.013), more proximal (46.1% vs. 33.3%, *P* = 0.029), and most proximal colon (34.2% vs. 13.1%, *P* < 0.001).

**Table 1:** Baseline characteristics and results of the screening colonoscopies in the control group and the periodontitis group.

	Control group (n = 2288)	Periodontitis group (n = 216)	*P* value^†^
Male sex	1269 (55.5%)	142 (65.7%)	0.004
Age, years	45.8 ± 10.8	51.9 ± 11.2	<0.001
Body mass index, kg/m^2^	23.7 ± 3.3	24.5 ± 3.3	0.001
Metabolic syndrome	238 (10.4%)	33 (15.3%)	0.027
Waist circumference, cm	84.3 ± 9.4	86.3 ± 8.8	0.002
Hypertension	662 (28.9%)	94 (43.5%)	<0.001
High fasting glucose	278 (12.2%)	52 (24.1%)	<0.001
Triglycerides, mg/dL	114.0 ± 77.7	120.0 ± 85.5	0.284
HDL, mg/dL	58.4 ± 15.7	54.8 ± 16.5	0.001
LDL, mg/dL	131.3 ± 34.3	128.9 ± 32.9	0.337
Total cholesterol, mg/dL	202.4 ± 36.1	200.2 ± 37.7	0.381
Smoking (ever)^*^	1003/2284 (43.9%)	131/216 (60.6%)	<0.001
Alcohol consumption^*^	1230/2284 (53.9%)	112/216 (51.9%)	0.573
FH of CRC	128 (5.6%)	16 (7.4%)	0.274
Aspirin use	65 (2.8%)	15 (6.9%)	0.001
Fatty liver	746 (32.6%)	78 (36.1%)	0.294
Physical activity	1337 (58.4%)	123 (56.9%)	0.671
Diverticulosis	55 (2.4%)	10 (4.6%)	0.068
**Results of screening colonoscopy**			
Colorectal neoplasms (all)	502 (21.9%)	76 (35.2%)	<0.001
Proximal colorectal neoplasms	281 (12.3)	54 (25.0)	<0.001
Advanced colorectal neoplasms	34 (1.5%)	9 (4.2%)	0.004
Proximal advanced colorectal neoplasms	20 (0.9%)	7 (3.2%)	0.001

Variables shown are numbers (percentages) or expressed as the mean ± standard deviation. ^*^Some data are missing. ^†^Differences in categorical variables between groups were analyzed using Chi-square test or Fisher’s exact test. Continuous variables were compared by Student’s t-test. Abbreviations: HDL, High-density lipoprotein; LDL, Low-density lipoprotein; FH of CRC, Family history of colorectal cancer.

**Table 2:** Clinicopathological characteristics of the colorectal neoplasms.

Characteristic	Control group (n = 502)	Periodontitis group (n = 76)	*P* value*
Number, n	1.4 ± 1.0	1.8 ± 1.0	0.009
1 or 2	457 (91.0%)	60 (78.9%)	0.001
≥3	45 (9.0%)	16 (21.1%)	
Size, mm	4.7 ± 3.6	5.7 ± 3.7	0.032
<5	301 (60.0%)	35 (46.1%)	0.015^†^
5–9	171 (34.1%)	33 (43.4%)	
≥10	30 (6.0%)	8 (10.5%)	
Low-grade dysplasia			0.284
Presence	496 (98.8%)	74 (97.4%)	
Absence	6 (1.2%)	2 (2.6%)	
High-grade dysplasia			1.000
Presence	4 (0.8%)	0 (0.0%)	
Absence	498 (99.2%)	76 (100.0%)	
Tubular adenoma			0.454
Presence	489 (97.4%)	73 (96.1%)	
Absence	13 (2.6%)	3 (3.9%)	
Tubulovillous/villous adenoma			0.232
Presence	5 (1.0%)	2 (2.6%)	
Absence	497 (99.0%)	74 (97.4%)	
Cancer			0.086
Presence	2 (0.4%)	2 (2.6%)	
Absence	500 (99.6%)	74 (97.4%)	
Advanced colorectal neoplasm			0.117
Presence	34 (6.8%)	9 (11.8%)	
Absence	468 (93.2%)	67 (88.2%)	
Appearance			0.001
Flat/depressed lesion	157 (31.3%)	10 (13.2%)	
Polypoid lesion	345 (68.7%)	66 (86.8%)	

Variables shown are numbers (percentages) or expressed as the mean ± standard deviation. *Differences in the categorical variables between the groups were analyzed using Chi-square test or Fisher’s exact test. Continuous variables were compared by Student’s t-test. ^†^linear by linear association X^2^ test.

**Table 3:** Clinicopathological characteristics (location) of the colorectal neoplasms.

Characteristic	Control group (n = 502)	Periodontitis group (n = 76)	*P* value*
**Location 1**			
Presence of CRNs in cecum			0.618
Yes	32 (6.4%)	6 (7.9%)	
No	470 (93.6%)	70 (92.1%)	
Presence of CRNs in proximal AC			<0.001
Yes	36 (7.2%)	23 (30.3%)	
No	466 (92.8%)	53 (69.7%)	
Presence of CRNs in distal AC			0.032
Yes	80 (15.9%)	5 (6.6%)	
No	422 (84.1%)	71 (93.4%)	
Presence of CRNs in HF			0.440
Yes	34 (6.8%)	7 (9.2%)	
No	468 (93.2%)	69 (90.8%)	
Presence of CRNs in TC			0.026
Yes	130 (25.9%)	29 (38.2%)	
No	372 (74.1%)	47 (61.8%)	
Presence of CRNs in SF			0.131
Yes	0 (0.0%)	1 (1.3%)	
No	502 (100.0%)	75 (98.7%)	
Presence of CRNs in DC to rectum			0.174
Yes	299 (59.6%)	39 (51.3%)	
No	203 (40.4%)	37 (48.7%)	
**Location 2**			
Presence of CRNs in proximal colon			0.013
Yes	281 (56.0%)	54 (71.1%)	
No	221 (44.0%)	22 (28.9%)	
Presence of CRNs in more proximal colon			0.029
Yes	167 (33.3%)	35 (46.1%)	
No	335 (66.7%)	41 (53.9%)	
Presence of CRNs in most proximal colon			<0.001
Yes	66 (13.1%)	26 (34.2%)	
No	436 (86.9%)	50 (65.8%)	

Variables shown are numbers (percentages). *Differences in the categorical variables between the groups were analyzed using Chi-square test or Fisher’s exact test. Abbreviations: CRNs, colorectal neoplasms; Proximal AC, Proximal half of the ascending colon; Distal AC, Distal half of the ascending colon; HF, Hepatic flexure; TC, Transverse colon; SF, Splenic flexure; DC, Descending colon.

### Risk factors for colorectal neoplasms according to location

The univariate analyses of the risk factors for CRNs according to the location (all CRNs, proximal CRNs, more proximal CRNs, and most proximal CRNs) are shown in Supplementary Table 1. There were significant differences between the subjects with and without CRNs (all) with respect to 15 factors (periodontitis, male, age, BMI, metabolic syndrome, waist circumference, hypertension, high fasting glucose, triglycerides, HDL, smoking, FH of CRC, aspirin use, fatty liver, and tooth loss). In addition, there were significant differences between the subjects with and without proximal CRNs with respect to 16 factors (15 factors same as above and cavities). After performing a univariate analysis, 13 factors (periodontitis, male, age, BMI, waist circumference, hypertension, high fasting glucose, triglycerides, HDL, smoking, FH of CRC, aspirin use, and tooth loss) were significantly associated with an increased risk of more proximal CRNs. In the case of most proximal CRNs, we found that 11 factors (periodontitis, age, waist circumference, hypertension, high fasting glucose, HDL, smoking, FH of CRC, aspirin use, fatty liver, and tooth loss) were associated with an increased risk of most proximal CRNs.

Table 4 presents the results of the multivariate logistic regression analysis of the risk factors for CRNs according to the location (all CRNs, proximal CRNs, more proximal CRNs, and most proximal CRNs). From the analysis of all CRNs, age (OR 1.063, *P* < 0.001), male (OR 1.915, *P* < 0.001), waist circumference (OR 1.014, *P* = 0.023), High fasting glucose (OR 1.403, *P* = 0.013), and FH of CRC (OR 1.514, *P* = 0.035) were significant independent risk factors for all CRNs. In the case of proximal CRNs, the risk factors were periodontitis (OR 1.525, *P* = 0.019), smoking (OR 1.700, *P* < 0.001), age (OR 1.071, *P* < 0.001), waist circumference (OR 1.022, *P* = 0.003), and triglycerides (OR 1.002, *P* = 0.023). In the case of more proximal CRNs, the risk factors were periodontitis (OR 1.598, *P* = 0.027), smoking (OR 1.600, *P* = 0.003), age (OR 1.074, *P* < 0.001), and triglycerides (OR 1.002, *P* = 0.035). For most proximal CRNs, periodontitis (OR 3.145, *P* < 0.001), smoking (OR 1.600, *P* = 0.036), age (OR 1.066, *P* < 0.001), and FH of CRC (OR 2.043, *P* = 0.039) were the independent risk factors. Furthermore, limiting the analysis to the periodontitis group (n = 216), significant risk factors for proximal CRNs were age (OR 1.088, P < 0.001) and male (OR 3.259, P = 0.004) (Supplementary Tables 2 and 3).

**Table 4:** Multivariate analysis of risk factors for colorectal neoplasms according to location, advanced colorectal neoplasms and proximal advanced colorectal neoplasms among the whole study group (including control).

Variable	*P* value	Odds ratio	95% confidence interval	
			Lower	Upper
**All CRNs (Cecum to rectum)**				
Age	<0.001	1.063	1.053	1.073
Male	<0.001	1.915	1.522	2.410
Waist circumference	0.023	1.014	1.002	1.027
High fasting glucose	0.013	1.403	1.073	1.834
FH of CRC	0.035	1.514	1.030	2.224
**Proximal CRNs (Cecum to SF)**				
Periodontitis	0.019	1.525	1.071	2.172
Smoking (ever)	<0.001	1.700	1.307	2.210
Age	<0.001	1.071	1.059	1.083
Waist circumference	0.003	1.022	1.007	1.037
Triglycerides	0.023	1.002	1.000	1.003
**More proximal CRNs (Cecum to HF)**				
Periodontitis	0.027	1.598	1.055	2.420
Smoking (ever)	0.003	1.600	1.173	2.184
Age	<0.001	1.074	1.059	1.089
Triglycerides	0.035	1.002	1.000	1.003
**Most proximal CRNs (Cecum to Proximal AC)**				
Periodontitis	<0.001	3.145	1.908	5.184
Smoking (ever)	0.036	1.600	1.032	2.482
Age	<0.001	1.066	1.045	1.087
FH of CRC	0.039	2.043	1.035	4.032
**Advanced colorectal neoplasms**				
Age	<0.001	1.055	1.028	1.083
Waist circumference	0.012	1.042	1.009	1.076
**Proximal advanced colorectal neoplasms**				
Periodontitis	0.032	2.671	1.088	6.560
Age	0.002	1.056	1.020	1.093
FH of CRC	0.022	3.215	1.185	8.725

Abbreviations: CRNs, Colorectal neoplasms; FH of CRC, Family history of colorectal cancer; SF, Splenic flexure; HF, Hepatic flexure; Proximal AC, Proximal half of the ascending colon.

### Risk factors for advanced colorectal neoplasms and proximal advanced colorectal neoplasms

We then evaluated the predictive factors associated with an increased risk of advanced CRNs and proximal advanced CRNs (Supplementary Table 4 and Table 4). From the multivariate logistic regression analysis, age (OR 1.055, P < 0.001) and waist circumference (OR 1.042, P = 0.012) were the significant predictive factors for advanced CRNs. For proximal advanced CRNs, the significant independent predictive factors were periodontitis (OR 2.671, P = 0.032), age (OR 1.056, P = 0.002), and FH of CRC (OR 3.215, P = 0.022).

## Discussion

This cross-sectional study assessed the predictive factors for proximal CRNs and proximal advanced CRNs with respect to periodontitis. We found that subjects with periodontitis were at a significantly higher risk for the presence of proximal CRNs (OR 1.525) and proximal advanced CRNs (OR 2.671) compared to subjects without periodontitis, independent of age, sex, smoking, and other known risk factors for CRNs, although periodontitis was not a significant risk factor for the presence of overall CRNs and advanced CRNs.

Interval CRCs are more likely to occur in the proximal colon mainly due to the missed pre-malignant lesions^4,5,8^. Therefore, identifying predictive factors for premalignant lesions in the proximal colon could provide clinically valuable information for colonoscopists. Although previous studies have reported several factors predicting the presence of proximal CRNs or proximal advanced CRNs such as age, sex, smoking, distal adenoma, FH of CRC, hypertension, and BMI^14–17,39–41^, most of them are the same as the risk factors for overall CRNs^19^. Thus, there is still limited information on risk factors specific for CRNs in proximal lesions. In this study, for the first time, we identified periodontitis as a specific predictive factor for proximal CRNs and proximal advanced CRNs.

A recent study reported that moderate to severe periodontal disease might increase the risk of proximal colon cancer (hazard ratios 1.23), suggesting a potential role of oral health in colorectal carcinogenesis^20^. In our findings, 3 variables (periodontitis, age, and smoking) were the common independent risk factors for proximal, more proximal, and most proximal CRNs. Among these 3 risk factors, the odds ratio of periodontitis increased gradually from proximal CRNs to most proximal CRNs (OR 1.525 in proximal CRNs, OR 1.598 in more proximal CRNs, OR 3.145 in most proximal CRNs), while the odds ratio of the other 2 risk factors (age and smoking) did not appear to increase according to the location (Table 4). This suggests that periodontitis is more likely to be associated with the proximity to the cecum than the other 2 risk factors. In addition, our findings regarding the clinicopathological characteristics (location) of the CRNs showed that the proportion of subjects with CRNs detected in the proximal AC and transverse colon was significantly higher in the periodontitis group. In contrast, the proportion of subjects with CRNs in the DC to the rectum was not significantly different. Taken together, the above results all suggest that the association between periodontitis and CRNs might increase gradually from the distal to the proximal colon. Previous molecular and microbiome studies may support this finding. *Fusobacterium*, the most prevalent periodontal pathogen, has been known to promote colorectal carcinogenesis through various mechanisms such as recruitment of tumor-infiltrating immune cells, activation of the Wnt/β-catenin oncogenic pathway and the NF-κB proinflammatory pathway^30,42^. *Fusobacterium* is found at increased abundance in proximal CRNs, with a gradual increase in *Fusobacterium-*high CRCs from the rectum to the cecum^33–35^. *Fusobacterium* may have a role in the carcinogenesis of the proximal colon through the serrated neoplasia pathway^34^. In sessile serrated adenomas, *Fusobacterium* positivity increased gradually from the sigmoid colon to the cecum^35^. The increased *Fusobacterium* in proximal CRNs may be due to the anaerobic condition, colonic lumen contents, and bacterial biofilms (bacterial aggregates)^43–45^. Bacterial biofilms, which are suggested to correlate with bacterial tissue invasion with oncogenic transformation, have been found to be prevalent higher in the proximal tumor than in the distal tumor (89% vs. 12%)^44^. Interestingly, a gradual increase of *Fusobacterium-*high CRCs from the distal to the proximal colon coincides with the gradual increase of CIMP-high and MSI- high CRNs from the rectum to the ascending colon^33,36^. In this regard, investigators have shown the association of *Fusobacterium* with CIMP-high and MSI- high status, suggesting a potential role of *Fusobacterium* in the early stage of colorectal tumorigenesis in the proximal colon^35^. Moreover, other periodontitis related genera including *Leptotrichia, Campylobacter, Prevotella*, and *Bacteroides* were reported to be associated with CRC, suggesting that an imbalance in the gut microbiota has a role in colorectal carcinogenesis^21,27,29,46^. Among these, in particular, *Prevotella* was reported to be highly enriched in proximal colon cancer^46^. Taken together, these molecular and microbiome findings indicate that periodontitis might be involved in the carcinogenesis in the proximal colon via periodontal pathogen related gut dysbiosis^26,47^. However, biological data clarifying the mechanism underlying the linkage between periodontitis and proximal CRNs are still limited, and additional studies are needed to confirm our findings.

In addition to periodontitis, our logistic analysis showed that smoking was another common independent risk factor for the presence of proximal, more proximal, and most proximal CRNs. This is consistent with previous reports that demonstrated the positive association between smoking and proximal CRNs^48–50^. Molecular studies have demonstrated a definitive link between smoking and colorectal carcinogenesis such as MSI, CIMP, and a serrated pathway, which occur most often in the proximal colon^48,49^. Meanwhile, smoking increases the abundance of periodontal pathogen including *Fusobacterium* and *Bacteroides*, as well as the severity of periodontitis^28,51,52^, which might explain another mechanism that smoking affects the development of proximal CRNs via periodontal pathogen related gut dysbiosis^26,47^.

In our findings shown in Table 1, the prevalence of overall CRNs and advanced CRNs was significantly higher in the periodontitis group than in the control group. However, the multivariate analysis showed that periodontitis was not an independent risk factor for overall CRNs and advanced CRNs. This is in agreement with previous studies which showed null associations between a history of periodontal disease and CRC risk^24,53,54^. In contrast, other studies demonstrated that there is a positive association between periodontal disease and CRNs^20,21,38^. A recent study showed that a history of periodontal disease with bone loss was not associated with CRC risk, although moderate to severe periodontal disease was a significant risk factor for CRC^20^. These contradictory findings may be explained by the self-reported history of periodontal disease which was prone to error and differences in the definition of subjects with periodontal disease.

There are some limitations to the current research. First, this study was conducted with a cross-sectional design. Therefore, there may be uncertainty about the causal relationship. Second, there is a possibility of selection bias because the subjects were recruited from individuals who visited the hospital for health check-ups and were more concerned about their health status. Moreover, the mean age of the study subjects (46.3 years) was relatively young, which may explain the relatively low prevalence of periodontitis in this study (8.6%) compared to previous studies which reported the prevalence of periodontitis ranges from 14% to 82%^38,55,56^. Third, we were unable to collect other objective indicators of periodontitis such as PPD, clinical attachment loss, radiologic bone loss, and bleeding on probing, which are required to evaluate the relationship between the severity of periodontitis and proximal CRNs. Fourth, data about the duration of periodontitis were not considered in the definition of the periodontitis group. The duration of periodontitis may be an important factor to determine whether periodontitis affects the development of proximal CRNs. Finally, we were unable to check the inter-examiner agreement for the diagnosis of periodontitis. However, considering the extensive experience of the 2 dentists involved and the simple definition of periodontitis used in this study, any associated bias should be minimal.

In conclusion, individuals with periodontitis might be at increased risk of proximal CRNs and proximal advanced CRNs. Therefore, colonoscopists should perform a more meticulous inspection of the proximal colon in subjects with periodontitis.

## Methods

### Ethical approval and informed consent

This study was approved by Institutional Review Board (IRB) of the CHA Bundang Medical Center (Approval Number: CHAMC 2017-07-036). All methods were performed in accordance with the relevant guidelines and regulations by the IRB. All subjects provided written informed consent for participation in this study.

### Study population

This was a cross-sectional, retrospective study that reviewed the medical records of subjects who underwent colonoscopy as a part of routine health check-ups from January to September 2016 at the CHA Bundang medical center, Korea. A total of 2633 subjects who received a colonoscopy and a dental exam and filled out a standard questionnaire were enrolled. We excluded subjects that had any of the following: (1) Colonoscopy for any reason in the previous 5 years; (2) History of colorectal cancer or polyp; (3) History of colorectal surgery or polypectomy; (4) History of inflammatory bowel disease or intestinal tuberculosis; (5) Incomplete colonoscopy (poor bowel preparation or cecal intubation failure) or missing data on the colonoscopy; (6) Failure of an adequate biopsy; (7) Incomplete dental exam or missing data on the dental exam; (8) denture status

### Dental examination

All dental examinations were performed by 2 dentists with over 10 years of extensive experience. The presence of dental cavities and tooth loss were recorded. Probing pocket depth (PPD) was assessed using a periodontal probe at six sites (mesio-buccal, mid-buccal, disto-buccal, disto-lingual, mid-lingual and mesio-lingual) per tooth. The periodontitis group was defined as subjects who had one or more teeth with a PPD ≥4 mm.

### Detection of colorectal neoplasms

All colonoscopies were performed by 4 experienced gastroenterologists with specialty certificates in gastroenterology and endoscopy. Bowel preparation was performed with 2 L of polyethylene glycol with ascorbate (CM Light Power®; CMG Pharmaceuticals, Seoul, South Korea). The number, size, location, appearance of the CRNs, and the presence of diverticulosis were recorded. Polyp size was estimated by using open biopsy forceps. The appearance of a CRN was classified as either polypoid or flat/depressed. A flat/depressed lesion was defined as an endoscopically visible mucosal lesion with a height less than half the diameter of the lesion. Proximal CRNs were defined as CRNs which were detected in the proximal colon (cecum to splenic flexure [SF]). More proximal and most proximal CRNs were defined as those found in the more proximal colon (cecum to hepatic flexure [HF]) and in the most proximal colon (cecum to proximal half of the ascending colon [proximal AC]), respectively. For subjects with multiple CRNs in both the proximal colon and another location (descending colon [DC] to rectum), they were assigned to those with the presence of proximal CRNs. For subjects with multiple CRNs in both the more proximal colon and another location (Transverse colon [TC] to rectum), they were assigned to those with the presence of more proximal CRNs. For subjects with multiple CRNs in both the most proximal colon and another location (distal half of the ascending colon [distal AC] to rectum), they were assigned to those with the presence of most proximal CRNs. An advanced CRN was defined as a cancer or adenoma that satisfied any of the following: (1) at least 10 mm in diameter; (2) high-grade dysplasia; (3) a villous or tubulovillous component. A proximal advanced CRN was defined as an advanced CRN that was detected in the proximal colon (cecum to SF). For subjects with multiple CRNs, the size and appearance of the neoplasms with advanced pathology or the largest polyp were reported.

### Measurements, definitions, and laboratory assays

The subjects’ height, body weight and waist circumference were measured by a trained nurse. BMI was calculated as weight divided by height squared (kg/m^2^). Metabolic syndrome was defined based on the updated National Cholesterol Education Program/Adult Treatment Panel III criteria^57^. Laboratory tests, including serum glucose, triglycerides, high-density lipoprotein (HDL), low-density lipoprotein (LDL), and total cholesterol were measured after a fasting period of at least 12 hours on the day of the colonoscopy. Abdominal ultrasonography was used to determine the presence of a fatty liver.

### Questionnaire

All participants were asked to complete a questionnaire which included the following items: smoking status (ever, never), alcohol consumption, physical activity, FH of CRC in first-degree relatives, aspirin use (confirmed prescription in the medial record) and current medications (diabetes, hypertension). Participants receiving antihypertensive medication were included in the hypertension group. Participants receiving diabetes treatment or those with a high fasting blood glucose (≥110 mg/dL) were included in the high fasting glucose group^58^.

### Statistical analysis

All statistical analyses were done with SPSS version 22 for Windows (SPSS Inc., Chicago, Illinois, USA). Differences in categorical variables between groups were analyzed using Chi-square test or Fisher’s exact test or linear by linear association X^2^ test when required. Continuous variables were compared by Student’s t-test. All risk factors with a significant difference, as determined by univariate analysis, were included in the multivariate analysis by logistic regression. Odds ratios (OR) and 95% confidence intervals (CI) were calculated for each variable for multivariate analysis. *P* values < 0.05 were considered statistically significant.

### Ethics approval and consents to participate

This study was approved by Institutional Review Board of the CHA Bundang Medical Center (Approval Number: CHAMC 2017-07-036).

## Supplementary information

Supplementary table S1-S4
