# Supplementary material for: Periodontitis is associated with an increased risk for proximal colorectal neoplasms
Source: Sci Rep. 2019 May 17;9:7528. doi: 10.1038/s41598-019-44014-8 (PMC6525177; doi:10.1038/s41598-019-44014-8)
Supplement: Supplementary file 1 — Supplementary table S1-S4 [file 41598_2019_44014_MOESM1_ESM.pdf]

Periodontitis is associated with an increased risk for proximal colorectal neoplasms

Gun Woo Kim[1], Young Sang Kim[2], Soo Hyun Lee[2], Seung Geon Park[2], Duk Hwan Kim[1], Joo Young Cho[1], Ki Baik Hahm[1], Sung Pyo Hong[1], and Jun Hwan Yoo[1*]

Supplementary Table legends

Supplementary Table S1. Univariate analysis of risk factors for colorectal neoplasms according to location in the whole study group (including control). Variables shown are numbers (percentages) or expressed as the mean ± standard deviation. *Some data are missing. †Differences in the categorical variables between the groups were analyzed using Chi-square test or Fisher's exact test. Continuous variables were compared by Student's t-test. Abbreviations: HDL, High-density lipoprotein; LDL, Low-density lipoprotein; FH of CRC, Family history of colorectal cancer; CRNs, Colorectal neoplasms; SF, Splenic flexure; HF, Hepatic flexure; Proximal AC, Proximal half of the ascending colon.

Supplementary Table S2. Univariate analysis of risk factors for proximal colorectal neoplasms (cecum to SF) in the periodontitis group. Variables shown are numbers (percentages) or expressed as the mean ± standard deviation. *Some data are missing. †Differences in the categorical variables between the groups were analyzed using Chi-square test or Fisher's exact test. Continuous variables were compared by Student's t-test. Abbreviations: HDL, High-density lipoprotein; LDL, Low-density lipoprotein; FH of CRC, Family history of colorectal cancer; SF,

Splenic flexure.

Supplementary Table S3. Multivariate analysis of risk factors for proximal colorectal neoplasms (cecum to SF) in the periodontitis group. Abbreviations: SF, Splenic flexure.

Supplementary Table S4. Univariate analysis of risk factors for advanced colorectal neoplasms and proximal advanced colorectal neoplasms in the whole study group (including control). Variables shown are numbers (percentages) or expressed as the mean ± standard deviation. *Some data are missing. †Differences in the categorical variables between the groups were analyzed using Chi-square test or Fisher's exact test. Continuous variables were compared by Student's t-test. Abbreviations: HDL, High-density lipoprotein; LDL, Low-density lipoprotein; FH of CRC, Family history of colorectal cancer.

Supplementary Table S1.

Variable	All CRNs (Cecum to rectum)		*P* value†	Proximal CRNs (Cecum to SF)		*P* value†	More proximal CRNs (Cecum to HF)		*P* value†	Most proximal CRNs (Cecum to Proximal AC)		*P* value†
	Absence (n=1926)	Presence (n=578)		Absence (n=2169)	Presence (n=335)		Absence (n=2302)	Presence (n=202)		Absence (n=2412)	Presence (n=92)	
Periodontitis	140 (7.3)	76 (13.1)	<0.001	162 (7.5)	54 (16.1)	<0.001	181 (7.9)	35 (17.3)	<0.001	190 (7.9)	26 (28.3)	<0.001
Male sex	1017 (52.8)	394 (68.2)	<0.001	1179 (54.4)	232 (69.3)	<0.001	1282 (55.7)	129 (63.9)	0.025	1351 (56.0)	60 (65.2)	0.081
Age, years	44.6±10.4	51.9±10.9	<0.001	45.2±10.6	53.5±10.9	<0.001	45.6±10.7	54.4±10.5	<0.001	46.0±10.9	55.0±10.3	<0.001
Body mass index, kg/m^2	23.6±3.4	24.5±3.1	<0.001	23.7±3.4	24.6±3.1	<0.001	23.8±3.4	24.3±3.0	0.019	23.8±3.3	24.3±3.0	0.147
Metabolic syndrome	185 (9.6)	86 (14.9)	<0.001	220 (10.1)	51 (15.2)	0.005	244 (10.6)	27 (13.4)	0.225	260 (10.8)	11 (12.0)	0.721
Waist circumference, cm	83.8±9.4	86.6±8.9	<0.001	84.1±9.4	87.2±8.7	<0.001	84.3±9.4	86.2±8.6	0.004	84.4±9.4	86.7±8.7	0.022
Hypertension	519 (26.9)	237 (41.0)	<0.001	606 (27.9)	150 (44.8)	<0.001	665 (28.9)	91 (45.0)	<0.001	713 (29.6)	43 (46.7)	<0.001
High fasting glucose	201 (10.4)	129 (22.3)	<0.001	247 (11.4)	83 (24.8)	<0.001	280 (12.2)	50 (24.8)	<0.001	304 (12.6)	26 (28.3)	<0.001
Triglycerides, mg/dL	111.4±74.9	125.0±88.4	0.001	112.4±75.0	128.5±96.4	0.004	113.6±76.9	125.5±93.3	0.039	114.1±77.8	126.3±91.8	0.144
HDL, mg/dL	58.9±16.0	55.5±14.8	<0.001	58.6±16.0	54.8±14.1	<0.001	58.3±15.9	55.9±14.1	0.037	58.3±15.9	54.5±12.5	0.006
LDL, mg/dL	131.0±33.6	131.5±35.8	0.746	131.2±33.8	130.5±36.3	0.722	131.2±34.0	129.9±36.0	0.605	131.0±34.1	133.1±35.0	0.565
Total cholesterol, mg/dL	202.4±35.4	201.6±39.0	0.637	202.6±35.8	200.1±39.0	0.256	202.4±36.1	200.0±37.5	0.373	202.3±36.2	201.6±36.6	0.872
Smoking (ever)*	805/1924 (41.8)	329/576 (57.1)	<0.001	934/2167 (43.1)	200/333 (60.1)	<0.001	1019/2300 (44.3)	115/200 (57.5)	<0.001	1082/2410 (44.9)	52/90 (57.8)	0.016
Alcohol consumption*	1020/1924 (53.0)	322/576 (55.9)	0.223	1152/2167 (53.2)	190/333 (57.1)	0.184	1240/2300 (53.9)	102/200 (51.0)	0.428	1298/2410 (53.9)	44/90 (48.9)	0.353
FH of CRC	97 (5.0)	47 (8.1)	0.005	115 (5.3)	29 (8.7)	0.014	125 (5.4)	19 (9.4)	0.020	133 (5.5)	11 (12.0)	0.009
Aspirin use	49 (2.5)	31 (5.4)	0.001	56 (2.6)	24 (7.2)	<0.001	67 (2.9)	13 (6.4)	0.006	71 (2.9)	9 (9.8)	<0.001
Fatty liver	588 (30.5)	236 (40.8)	<0.001	675 (31.1)	149 (44.5)	<0.001	745 (32.4)	79 (39.1)	0.050	782 (32.4)	42 (45.7)	0.008
Physical activity	1132 (58.8)	328 (56.7)	0.386	1261 (58.1)	199 (59.4)	0.662	1339 (58.2)	121 (59.9)	0.632	1402 (58.1)	58 (63.0)	0.348
Diverticulosis	48 (2.5)	17 (2.9)	0.552	54 (2.5)	11 (3.3)	0.395	56 (2.4)	9 (4.5)	0.083	60 (2.5)	5 (5.4)	0.081
Tooth loss	123 (6.4)	66 (11.4)	<0.001	144 (6.6)	45 (13.4)	<0.001	160 (7.0)	29 (14.4)	<0.001	166 (6.9)	23 (25.0)	<0.001
Cavities	199 (10.3)	52 (9.0)	0.348	228 (10.5)	23 (6.9)	0.039	234 (10.2)	17 (8.4)	0.427	242 (10.0)	9 (9.8)	0.937

Supplementary Table S2.

Variable	Proximal colorectal neoplasms (Cecum to SF)		*P* value†
	Absence (n=162)	Presence (n=54)	
Male sex	99 (61.1)	43 (79.6)	0.013
Age, years	49.8±11.0	58.2±9.3	<0.001
Body mass index, kg/m^2	24.4±3.4	24.9±3.1	0.340
Metabolic syndrome	24 (14.8)	9 (16.7)	0.743
Waist circumference, cm	86.0±9.2	87.4±7.2	0.327
Hypertension	64 (39.5)	30 (55.6)	0.057
High fasting glucose	34 (21.0)	18 (33.3)	0.066
Triglycerides, mg/dL	117.6±81.7	127.4±96.7	0.468
HDL, mg/dL	55.7±17.6	52.2±12.4	0.108
LDL, mg/dL	129.5±33.8	127.1±30.1	0.641
Total cholesterol, mg/dL	201.6±39.1	195.8±33.0	0.322
Smoking (ever)*	94 (58.0)	37 (68.5)	0.172
Alcohol consumption*	78 (48.1)	34 (63.0)	0.059
FH of CRC	11 (6.8)	5 (9.3)	0.549
Aspirin use	9 (5.6)	6 (11.1)	0.164
Fatty liver	51 (31.5)	27 (50.0)	0.014
Physical activity	92 (56.8)	31 (57.4)	0.937
Diverticulosis	10 (6.2)	0 (0.0)	0.070
Tooth loss	144 (88.9)	45 (83.3)	0.285
Cavities	47 (29.0)	8 (14.8)	0.047

Supplementary Table S3.

Variable	*P* value	Odds ratio	95% confidence interval	
			Lower	Upper
**Proximal colorectal neoplasms (Cecum to SF)**				
Age	<0.001	1.088	1.051	1.127
Male	0.004	3.259	1.474	7.207

Supplementary Table S4.

Variable	Advanced colorectal neoplasms		*P* value†	Proximal advanced colorectal neoplasms		*P* value†
	Absence (n=2461)	Presence (n=43)		Absence (n=2477)	Presence (n=27)	
Periodontitis	207 (8.4)	9 (20.9)	0.004	209 (8.4)	7 (25.9)	0.001
Male sex	1386 (56.3)	25 (58.1)	0.811	1396 (56.4)	15 (55.6)	0.933
Age, years	46.2±10.9	53.3±11.1	<0.001	46.2±10.9	54.2±11.0	<0.001
Body mass index, kg/m^2	23.8±3.3	24.9±3.3	0.027	23.8±3.3	24.5±2.7	0.291
Metabolic syndrome	261 (10.6)	10 (23.3)	0.008	267 (10.8)	4 (14.8)	0.526
Waist circumference, cm	84.4±9.4	88.2±8.4	0.009	84.5±9.4	86.4±6.8	0.288
Hypertension	733 (29.8)	23 (53.5)	0.001	741 (29.9)	15 (55.6)	0.004
High fasting glucose	319 (13.0)	11 (25.6)	0.015	325 (13.1)	5 (18.5)	0.410
Triglycerides, mg/dL	114.2±78.4	132.9±78.5	0.123	114.5±78.5	121.6±67.2	0.638
HDL, mg/dL	58.2±15.9	55.4±12.1	0.143	58.2±15.8	53.3±12.2	0.049
LDL, mg/dL	131.1±34.2	132.2±30.6	0.829	131.0±34.2	138.1±27.2	0.285
Total cholesterol, mg/dL	202.2±36.3	203.1±33.5	0.871	202.2±36.3	205.3±25.1	0.656
Smoking (ever)*	1110/2457 (45.2)	24/43 (55.8)	0.165	1118/2473 (45.2)	16/27 (59.3)	0.145
Alcohol consumption*	1318/2457 (53.6)	24/43 (55.8)	0.777	1327/2473 (53.7)	15/27 (55.6)	0.844
FH of CRC	138 (5.6)	6 (14.0)	0.020	139 (5.6)	5 (18.5)	0.004
Aspirin use	80 (3.3)	0 (0.0)	0.401	80 (3.2)	0 (0.0)	1.000
Fatty liver	803 (32.6)	21 (48.8)	0.025	811 (32.7)	13 (48.1)	0.090
Physical activity	1437 (58.4)	23 (53.5)	0.518	1444 (58.3)	16 (59.3)	0.920
Diverticulosis	63 (2.6)	2 (4.7)	0.308	64 (2.6)	1 (3.7)	0.510
Tooth loss	181 (7.4)	8 (18.6)	0.006	183 (7.4)	6 (22.2)	0.004
Cavities	247 (10.0)	4 (9.3)	1.000	250 (10.1)	1 (3.7)	0.513
